# Efficacy of Madopar and trihexyphenidyl combination therapy for dystonia in children with cerebral palsy

**DOI:** 10.3389/fneur.2026.1707423

**Published:** 2026-01-26

**Authors:** Xiaolin Zhou, Xiangyang Luo, Zhanwen He, Mujin Liu, Pinggan Li

**Affiliations:** Department of Pediatric Neurology, Sun Yat-sen Memorial Hospital, Sun Yat-sen University, Guangzhou, China

**Keywords:** dyskinetic cerebral palsy, dystonia, Madopar, motorfunction, Trihexyphenidyl

## Abstract

**Introduction:**

Dystonia is a predominant and debilitating movement disorder associated with dyskinetic cerebral palsy (DCP). Although trihexyphenidyl (THP) is commonly used as a treatment, its efficacy often exhibits a plateau effect. The combination of dopaminergic and anticholinergic agents represents a rational therapeutic strategy; however, robust evidence for the combination of Madopar (levodopa/benserazide) and THP is lacking.

**Methods:**

This retrospective cohort study compared THP monotherapy (*n* = 25) with combined Madopar + THP therapy (*n* = 24) in children with DCP and dystonia. Propensity score matching was used to balance the baseline characteristics. Various outcomes were analyzed at baseline and at both 8 and 16 weeks, including the Barry-Albright Dystonia Scale (BADS), Gross Motor Function Measure-88 (GMFM-88), Quality of Upper Extremity Skills Test (QUEST), and Cerebral Palsy Quality of Life Questionnaire (CP-QOL) measures. Parent-reported improvements in daily activities, drooling, speech, and sleep were also analyzed.

**Results:**

Compared with the THP group, the Madopar + THP group demonstrated significantly greater reductions in dystonia severity at both 8 and 16 weeks (mean BADS change: −5.25 ± 1.45 vs. −2.52 ± 1.36 at 16 weeks, *p* < 0.001). Superior improvements were also observed in gross motor function (GMFM-88: 14.29 ± 3.39 vs. 8.56 ± 2.29), upper limb function (QUEST: 6.33 ± 1.43 vs. 3.24 ± 1.05), and quality of life (CP-QOL: 6.17 ± 2.12 vs. 3.24 ± 0.66, all *p* < 0.001). Notably, the combination therapy yielded markedly higher rates of parent-reported improvements in daily life (88% vs. 24%, *p* < 0.001) and easy of care (71% vs. 20%, *p* = 0.001) at 16 weeks. No serious adverse events were reported in either group.

**Discussion:**

Compared with THP monotherapy, the combination of Madopar and THP is significantly more effective at alleviating dystonia and improving both motor function and quality of life in children with DCP. By leveraging low-dose synergy, this strategy effectively overcomes the efficacy ceiling of first-line monotherapy and translates into meaningful, patient-centered functional gains (including improvements in sleep and communication) without increasing the burden of adverse events.

## 1 Introduction

Dystonia is the predominant and most debilitating movement disorder observed in patients with dyskinetic cerebral palsy (DCP) and severely impairs motor function, activities of daily living, and quality of life (1). First-line oral pharmacotherapy, including trihexyphenidyl (THP) and baclofen, provides incomplete relief for many children, with benefits often being limited by efficacy ceilings and side effects (2, 3).

THP is among the most frequently prescribed agents (4, 5). Although this drug can improve dystonia and motor function, a Cochrane review revealed insufficient high-quality evidence for its routine use, and partial efficacy is often observed regarding complex functional outcomes (6, 7). This scenario underscores the need for more effective treatment strategies.

Levodopa represents a distinct therapeutic pathway. Beyond its diagnostic role in rare dopa-responsive dystonia, emerging evidence suggests that sustained levodopa treatment may benefit children with acquired dystonia, such as that following hypoxic–ischemic injury common in DCP (8, 9). The proposed mechanism involves modulating dysfunctional dopaminergic pathways in the basal ganglia (10), and levodopa is clinically administered with a peripheral decarboxylase inhibitor (e.g., Madopar, levodopa/benserazide) to enhance central delivery and tolerability (11). Critically, given the well-established reciprocal interaction between dopamine and acetylcholine in regulating basal ganglia circuits and motor control (10), simultaneously targeting both systems via combination therapy—specifically, supplementing dopaminergic modulation with anticholinergic agents like trihexyphenidyl (THP)—represents a rational strategy. We hypothesize that this approach may yield synergistic effects to more effectively restore motor circuit balance and overcome the efficacy limitations of monotherapy. This rationale is preliminarily supported by evidence that combining THP with clonazepam outperforms THP alone in dystonic CP (12).

However, robust clinical evidence for the specific combination of an optimized levodopa formulation (Madopar) with THP in DCP remains lacking. Moreover, whether this specific combination offers synergistic efficacy that is superior to that of THP monotherapy (without compromising safety) remains a pivotal unanswered question. To address this issue, we conducted a retrospective cohort study directly comparing the efficacy and safety of Madopar + THP combination therapy and THP monotherapy in children with DCP and dystonia.

## 2 Methods

### 2.1 Study design and participants

This retrospective cohort study evaluated children with dyskinetic cerebral palsy (DCP) and concomitant dystonia who received either Madopar combined with trihexyphenidyl (THP) or THP monotherapy at the Department of Pediatric Neurology, Sun Yat-sen Memorial Hospital, between January 2023 and June 2024. Participants who met the diagnostic criteria for DCP with dystonia according to the Chinese Rehabilitation Guidelines for Cerebral Palsy (2022) (13) were aged 1–16 years, with no gender restrictions being utilized. The exclusion criteria included the use of other antidystonia medications (such as anticholinergics, baclofen, benzodiazepines, or related drugs) within 3 months prior to the study period; the receipt of botulinum toxin injections, surgical interventions, or other antidystonia treatments within 16 weeks before enrollment; a history of hypersensitivity to study medications; severe comorbidities unrelated to CP; treatment regimen changes during the study period (such as switching between monotherapy and combination therapy); or incomplete medical records. The study design ensured a homogeneous cohort for comparative analysis of treatment efficacy while controlling for potential confounding factors.

### 2.2 Treatment protocol and group allocation

This study did not involve any intervention or randomization of treatment regimens. A total of 49 children with cerebral palsy met the study criteria. Based on their actual treatment histories, the participants were divided into two groups: the Madopar + THP group (*n* = 24), which received combined Madopar (levodopa/benserazide) and THP therapy, and the THP group (*n* = 25), which received THP monotherapy. All of the participants had received their respective treatments for at least 6 months.

The Madopar + THP group received Madopar tablets [250 mg/tablet, containing 200 mg of levodopa and 50 mg of benserazide (equivalent to 57 mg of benserazide hydrochloride)] and THP tablets (2 mg/tablet). The initial Madopar dosage ranged from 5 to 10 mg/kg/day and was administered in 2–3 divided doses, with a maximum daily dose not exceeding 375 mg. THP was initiated at 0.1–0.4 mg/kg/day (2–3 divided doses), targeting a dose of 0.25 mg/kg/day with a maximum dose of 20 mg/day. Dosage adjustments (10–20% increments every 2 weeks) were performed according to tolerance; moreover, reductions were implemented when adverse effects occurred.

### 2.3 Outcome measures and assessments

To control for confounding bias, we performed 1:1 propensity score matching (PSM) to balance the baseline characteristics between the groups. Matching variables (selected according to cerebral palsy treatment guidelines and clinical practice) included age (±1 year), gender, CP subtype (exact match), GMFCS level (±1 level), GMFM-88 score (±1 point), and duration of prior rehabilitation (±3 months).

The outcome measures included the Barry-Albright Dystonia Scale (BADS), Gross Motor Function Measure-88 (GMFM-88), Quality of Upper Extremity Skills Test (QUEST), Cerebral Palsy Quality of Life Questionnaire (CP-QOL), and gross motor function classification, which were assessed at baseline, as well as at 8 weeks and 16 weeks after treatment. Parental reports on activities of daily living, drooling symptoms, speech improvement, and gross/fine motor functions were also reviewed. Although retrospective, all cases strictly followed our institution’s Clinical Pathway for dyskinetic cerebral palsy management, thus ensuring standardized treatment and assessment. The pathway mandates a comprehensive evaluation by a designated pediatric neurologist. Follow-up evaluations were conducted at 8 and 16 weeks.

### 2.4 Statistical analysis

All statistical analyses were conducted by using SPSS 25.0 software. The normality of the continuous variables was evaluated by using the Shapiro–Wilk test. Due to the fact that most of the data were not normally distributed (as shown in the results), the nonparametric Mann–Whitney *U* test was used for comparisons between the groups, and the Wilcoxon signed-rank test was used for comparisons within time points within the groups. Categorical data (frequency/percentage) were analyzed by using the *χ*^2^ test.

The Barry-Albright Dystonia Scale (BADS) and Gross Motor Function Measure-88 (GMFM-88) scores were prespecified as coprimary endpoints. Given the exploratory nature of the secondary outcomes, *p* values were reported without adjustment for multiple comparisons, and these results should be interpreted accordingly. Statistical significance was set at *p* < 0.05 (two-sided).

## 3 Results

### 3.1 Normality test of data

The Shapiro–Wilk test was used to assess the normality of the baseline values and change values of each scale. The results demonstrated that all of the measurement values of the BADS and the baseline values of the QUEST and GMFM-88 did not follow a normal distribution (all *p* < 0.05). Therefore, nonparametric test methods were adopted for the subsequent analysis.

### 3.2 Demographic data and baseline data

**Figure 1:**
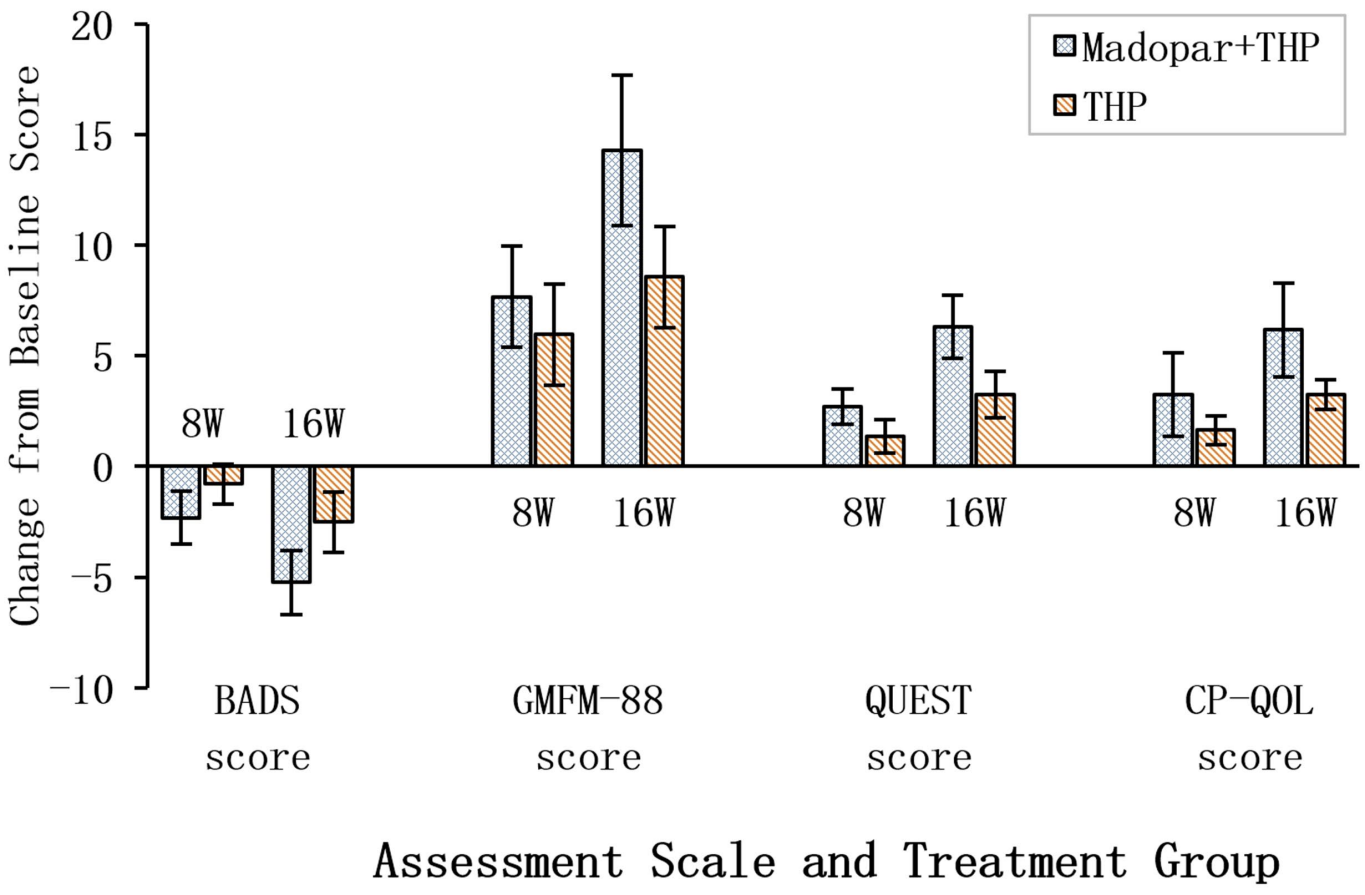
Comparison of treatment efficacy between Madopar + THP and THP monotherapy. Data are mean change from baseline ± SD. Negative values (bars extending left of the zero vertical line) indicate reduction in BADS score (improvement). Positive values (bars extending right) indicate increase in GMFM-88, QUEST, and CP-QOL scores (improvement). Side-by-side bars compare the two treatment groups at 8 and 16 weeks, *p* < 0.05.

A total of 49 children with dyskinetic cerebral palsy were enrolled and divided into two groups: the Madopar + THP group (*n* = 24) and the THP group (*n* = 25), with comparable baseline demographics and clinical characteristics being observed (Table 1). All of the participants were classified as GMFCS levels 2–4. Compared with baseline, both groups exhibited significant improvements in dystonia severity (BADS scores), gross motor function (GMFM-88), upper limb function (QUEST), and quality of life (CP-QOL) at 8 and 16 weeks (*p* < 0.05); however, the outcomes of the Madopar + THP group were superior. Specifically, at 8 and 16 weeks, the Madopar + THP group exhibited greater reductions in BADS scores (−2.33 ± 1.20 vs. −0.80 ± 0.91 and −5.25 ± 1.45 vs. −2.52 ± 1.36, respectively), greater improvements in GMFM-88 scores (7.67 ± 2.28 vs. 5.96 ± 2.30 and 14.29 ± 3.39 vs. 8.56 ± 2.29, respectively), greater gains in QUEST scores (2.71 ± 0.81 vs. 1.36 ± 0.76 and 6.33 ± 1.43 vs. 3.24 ± 1.05, respectively), and greater improvements in CP-QOL scores (3.25 ± 1.87 vs. 1.64 ± 0.64 and 6.17 ± 2.12 vs. 3.24 ± 0.66, respectively) compared to the THP group (*p* < 0.05; Table 2). These comparative improvements are visually summarized in Figure 1, which clearly demonstrates the greater efficacy of the combination therapy across both time points for all primary and secondary outcome measures.

**Table 1:** Comparison of baseline clinical and sociodemographic variables between groups.

Variable	Madopar + THP group (*N* = 24)	THP group (*N* = 25)	*p* value
Age (years)	3.4 ± 2.3	3.5 ± 2.0	0.762
Gender: male	17 (70.8%)	18 (72.0%)	1.000
GMFCS level (median, IQR)	3 (2–4)	3 (2–4)	0.720
Dose of THP (mg/kg/day)			
Average starting dose	0.23 ± 0.11	0.28 ± 0.12	0.193
Average maximum dose	0.48 ± 0.28	0.67 ± 0.29	0.073
Dose of Madopar (mg/kg/day)			
Average starting dose	8.26 ± 2.65	—	—
Average maximum dose	14.86 ± 7.69	—	—
BADS score	15.92 ± 2.71	16.40 ± 2.38	0.645
QUEST score	31.38 ± 8.35	34.40 ± 9.96	0.317
GMFM score	71.04 ± 27.06	78.28 ± 25.61	0.285
CP-QOL score	36.54 ± 7.95	35.72 ± 8.26	0.892

**Table 2:** Comparison of changes in primary and secondary efficacy outcome measures in both groups.

Change from baseline	Madopar + THP group (*N* = 24)	THP group (*N* = 25)	*p* value
BADS score			
8 weeks-baseline	−2.33 ± 1.20	−0.80 ± 0.91	<0.001
16 weeks-baseline	−5.25 ± 1.45	−2.52 ± 1.36	<0.001
GMFM-88 score			
8 weeks-baseline	7.67 ± 2.28	5.96 ± 2.30	0.003
16 weeks-baseline	14.29 ± 3.39	8.56 ± 2.29	<0.001
QUEST score			
8 weeks-baseline	2.71 ± 0.81	1.36 ± 0.76	<0.001
16 weeks-baseline	6.33 ± 1.43	3.24 ± 1.05	<0.001
CP-QOL score			
8 weeks-baseline	3.25 ± 1.87	1.64 ± 0.64	<0.001
16 weeks-baseline	6.17 ± 2.12	3.24 ± 0.66	<0.001

### 3.3 Therapeutic effects observed at 8 and 16 weeks

As shown in Table 3, both groups demonstrated significant improvements in dystonia severity, upper limb function, gross motor function, and quality of life at 8 and 16 weeks compared with baseline (*p* < 0.05), with the Madopar + THP group exhibiting significantly greater improvements than the THP group (*p* < 0.05). Notably, all of the measured parameters demonstrated more pronounced improvements at 16 weeks than at 8 weeks (*p* < 0.05; Table 3).

**Table 3:** Comparison of primary and secondary efficacy outcome measures within each group compared to baseline.

Madopar + THP group	At baseline (*N* = 24)	At 8 weeks (*N* = 24)	At 16 weeks (*N* = 24)	*p* value
BADS score	15.92 ± 2.71	13.58 ± 2.38	10.67 ± 1.81	<0.001
GMFM-88 score	71.04 ± 27.06	78.71 ± 27.25	85.33 ± 28.00	<0.001
QUEST score	31.38 ± 8.35	34.08 ± 8.62	37.71 ± 9.35	<0.001
CP-QOL score	36.54 ± 7.95	39.79 ± 6.81	42.71 ± 6.52	<0.001

THP group	At baseline (*N* = 24)	At 8 weeks (*N* = 24)	At 16 weeks (*N* = 24)	*p* value
BADS score	16.40 ± 2.38	15.60 ± 2.24	13.88 ± 1.79	<0.001
GMFM-88 score	78.28 ± 25.61	84.24 ± 25.24	92.48 ± 25.72	<0.001
QUEST score	34.40 ± 9.96	35.76 ± 9.95	37.64 ± 10.07	<0.001
CP-QOL score	35.72 ± 8.26	37.36 ± 8.36	38.96 ± 8.38	<0.001

### 3.4 Secondary outcomes and adverse effects of the two groups

Furthermore, children in the Madopar + THP group began demonstrating improvements in activities of daily living, salivation, dysarthria, and sleep disturbances after 8 weeks of treatment, with most patients demonstrating marked improvements by 16 weeks. In contrast, the THP group only exhibited significant improvement in salivation at 8 weeks, with no notable changes in communication ability, dysarthria, or sleep disturbances being observed. By 16 weeks, 67% of the caregivers in the THP group reported improved dysarthria, whereas the other aspects remained largely unchanged (Table 4).

**Table 4:** Secondary outcomes and adverse effects of two groups (16 weeks).

Outcome/adverse effect	Madopar + THP group (*N* = 24)	THP group (*N* = 25)	*p* value
Number of cases improved *n* (%)			
Salivation	5/5 (100%)	5/5 (100%)	1.000
Dysarthria	6/7 (86%)	4/6 (67%)	0.592
Sleep disorder	13/16 (81%)	4/15 (27%)	0.006
Daily life	21/24 (88%)	6/25 (24%)	<0.001
Communication ability	13/24 (54%)	5/25 (20%)	0.024
Ease of care	17/24 (71%)	5/25 (20%)	0.001
Adverse effects			
Constipation	1 (4%)	2 (8%)	1.000
Urinary retention	0 (0%)	1 (4%)	1.000
Emotional disorders	4 (17%)	3 (12%)	0.695
Sleep disorder	1 (4%)	2 (8%)	1.000
Dystonia	0 (0%)	0 (0%)	—
Gastrointestinal reactions	3 (13%)	2 (8%)	0.669
Rash	0 (0%)	0 (0%)	—
Thirsty	0 (0%)	0 (0%)	—
Incidence of adverse reactions	38%	40%	0.866

No serious adverse events occurred in either group. The most common adverse reaction detected in the Madopar + THP group included mood disturbances, followed by gastrointestinal symptoms. Similarly, the THP group most frequently experienced mood disturbances, along with sleep disorders, constipation, and gastrointestinal symptoms. For patients exhibiting excitement, irritability, or sleep disturbances, we adjusted dosing schedules by administering the final daily dose before 17:00 to minimize nocturnal effects. Mild adverse reactions such as decreased appetite, nausea, and constipation typically resolved within 3 days of continued medication. Importantly, no patients experienced intolerable adverse effects requiring treatment discontinuation.

## 4 Discussion

This study provides the first comparative evidence that the combination of Madopar (levodopa/benserazide) with trihexyphenidyl (THP) yields significantly greater improvements in dystonia severity, motor function, and quality of life in children with dyskinetic cerebral palsy (DCP) compared to THP monotherapy (without increasing the overall burden of adverse events). These findings validate a rational, pathophysiology-driven combination strategy and address critical gaps in both evidence and practice.

### 4.1 THP monotherapy: establishing the baseline and its limitations

Our results first reaffirm THP as an active agent in DCP, with the monotherapy group exhibiting significant improvements. These findings align with real-world data in which THP improves symptoms in many children (7, 14). However, the magnitude of the benefit from THP alone was modest, particularly in complex functional domains such as speech and sleep. This observation precisely mirrors the conclusion of a Cochrane systematic review that reported low-quality evidence for the efficacy of THP on standardized dystonia scales (6, 15), thus highlighting a clear efficacy ceiling. This gap between widespread clinical use (4, 16) and limited high-grade evidence underscores the urgent need for more effective strategies.

### 4.2 The therapeutic rationale for dopaminergic augmentation

The clear superiority of the combination therapy across all efficacy measures, as visually summarized in Figure 1, provides compelling support for the strategic use of dopaminergic augmentation in acquired dystonia. Although modern consensus advises against its empirical diagnostic use (17), emerging evidence indicates that long-term levodopa treatment may provide functional benefits in specific non-dopa-responsive dystonia populations (18–21). Children with DCP following hypoxic–ischemic injury represent a prime example of this scenario. Our findings suggest that supplementation of THP with low-dose Madopar may enhance dopaminergic signaling in compromised basal ganglia circuits, thereby facilitating motor adaptation (8, 22). The use of the benserazide-levodopa formulation (Madopar) was intentional to optimize central delivery and tolerability (11).

### 4.3 Synergy and the clinically vital “low-dose synergy” effect

The success of the combination treatment is attributed to the reciprocal dopamine-acetylcholine imbalance within basal ganglia circuits (10, 23). THP reduces excessive cholinergic tone, whereas levodopa supplements dopaminergic drive, thus suggesting synergy. Crucially, this process may explain a “low-dose synergy” effect. The THP doses utilized in our combination group were lower than those utilized in a large series using THP alone (24); however, they achieved superior efficacy. This finding indicates that the combination can achieve greater benefit without increasing THP doses into ranges associated with intolerable anticholinergic side effects (6, 25), which is a common reason for treatment failure.

### 4.4 Functional impact on patient-centered outcomes

The most striking benefits were observed in the parent-reported outcomes, including dramatic improvements in sleep (88% vs. 24%) and communication. These domains are minimally affected by monotherapy but are paramount treatment goals for caregivers and clinicians alike (26–28). Our study demonstrates that effective dystonia management can translate into these meaningful, real-world gains.

### 4.5 Limitations and future directions

This study has limitations that are intrinsic to its retrospective design. First, despite the use of propensity score matching, nonrandomized treatment allocation may lead to residual confounding risks, as clinical factors influencing the choice of combination therapy (e.g., perceived dystonia complexity) may not be fully captured. Second, multiple statistical comparisons for secondary outcomes increase the risk of type I error; therefore, these results should be considered hypothesis-generating. Third, the small sample size limits subgroup analyses and the detection of rare adverse events. Finally, the 16-week follow-up is insufficient for assessing long-term efficacy, tolerance, and safety. These limitations precisely demonstrate the need for future research involving the performance of a large-scale, randomized, double-blind, placebo-controlled trial with longer follow-up, which is essential for confirmation. Our current findings provide robust preliminary evidence supporting the use of the Madopar-THP combination as a superior and viable clinical strategy when THP monotherapy is inadequate.

## 5 Conclusion

In conclusion, the combination of Madopar and THP is a more effective strategy than THP monotherapy for children with DCP and dystonia. This rational polypharmacy approach overcomes the efficacy ceiling of first-line monotherapy, delivers critical functional improvements through low-dose synergy, and maintains a tolerable safety margin. Our findings provide a strong rationale for further investigations and cautious clinical application.

## Data Availability

The original contributions presented in the study are included in the article/Supplementary material, further inquiries can be directed to the corresponding author.
